# Prevalence of active hepatitis C virus infections among general public of Lahore, Pakistan

**DOI:** 10.1186/1743-422X-10-351

**Published:** 2013-12-05

**Authors:** Muhammad Ikram Anwar, Moazur Rahman, Mahmood Ul Hassan, Mazhar Iqbal

**Affiliations:** 1 Health Biotechnology Division, National Institute for Biotechnology and Genetic Engineering (NIBGE), Jhang Road, P. O. BOX 577, Faisalabad 38000, Pakistan; 2 Department of Statistics, Stockholm University, Stockholm, SE 106 91, Sweden

**Keywords:** Active HCV prevalence, Lahore, Pakistan, Nested PCR

## Abstract

**Background:**

To find out the prevalence of active hepatitis C virus (HCV) infections among general public in Lahore city, since data concerning the prevalence of active HCV in this city is currently unavailable.

**Methods:**

Blood samples were collected randomly from individuals visiting different clinical laboratories in Lahore. Serum was separated and processed by nested PCR qualitative assay for the detection of HCV RNA. The samples were categorized into different age groups on the basis of pre-test questionnaires in order to record the age-wise differences regarding the prevalence of active HCV. Data were analyzed statistically using Chi-Square test.

**Results:**

Out of the 4246 blood samples analyzed in this study, 210 were confirmed to be positive for active HCV infection. Gender-wise active HCV prevalence revealed no significant difference [OR = 1.10 CI = (0.83-1.46), *p* > 0.05]. However, among the age groups the highest prevalence was observed in the age groups 20–29 (7.7%) and 30–39 years (6.4%) with odds of prevalence of 14.8% (OR = 2.48, CI = (1.40-4.38), *p* < 0.05) and 10.3% (OR = 2.03, CI = (1.10-3.71), respectively. In age groups above 40 years (40–49, 50–59 and >59 years), a decrease in levels of active HCV prevalence was observed.

**Conclusions:**

Among tested samples, 4.9% of the subjects were confirmed to harbour active HCV infections and the “middle aged” population in Lahore was found to be at a higher risk of the HCV ailments compared to both their younger and older peers.

## Background

Hepatitis C is an infectious liver disease of humans and chimpanzees and is caused by the HCV [1]. The infection is often asymptomatic especially in its early stages but once established, it can progress to advanced liver diseases such as liver fibrosis and ultimately cirrhosis. These liver diseases can further lead to other complications such as liver failure and liver cancer [2]. In 2004, the World Health Organization (WHO) reported that annual deaths all over the world due to liver cancer and cirrhosis caused by HCV were about 308,000 and 785,000, respectively [3]; and about 200 million people, the 3.3% of the world’s population, are infected with HCV [4]. Moreover, around 3 to 4 million individuals are diagnosed as new cases every year [5].

In Pakistan an alarming rate of HCV outbreaks have been reported. The previous estimates indicate that around 10 million people are infected with HCV in Pakistan [6-8]. Prevalence of HCV has been noticed to be highly variable in different regions and even in different groups of the same community [9]. According to various studies, the presence of HCV infections among different categories (excluding chronic liver disease patients), was 5.31% in Islamabad [10], 0.4-31.9% in various regions of Punjab province [6,7,11-13], 4-6% in Sindh province [6,7,13,14], 1.1-9% in Khyber Pakhtunkhwa province [6,7,13,15-17], 1.5% in Quetta region [12,13] and 25.7% in Gilgit Baltistan province [18,19]. While in Lahore, the second largest city of Pakistan with a population of more than 7 million [20], HCV prevalence was estimated from 0.58-17.78% [6,13,19,21-24].

Two major issues undermine the credibility of the published data: firstly, the number of samples included in most of the cohort studies were too small; secondly, dissimilar methodologies adapted by various researchers made it highly inappropriate to conduct a formal meta-analysis to assess the accurate national prevalence [7]. Most of the studies conducted so far, have relied on the presence of anti-HCV antibodies in the blood samples using immunochromatographic tests (ICT), enzyme immunoassay (EIA), recombinant strip immunosorbent assay (RIBA) and enzyme linked immunosorbent assay (ELISA) techniques. All these techniques are fairly error prone and have been reported to generate around 50% false positive results as compared to PCR confirmatory assay [15]. Approximately 60-85% of HCV patients can develop chronic hepatitis, while the remaining 15-40% can clear the HCV infection. A number of these individuals, however, can still be detected as HCV positive using non-PCR immunoassays such as ELISA, RIBA, EIA, ICT etc., due to the presence of anti-HCV antibodies in their blood [15]. Nevertheless, to date there is hardly any PCR based prevalence studies conducted in Pakistan with statistically representative number of samples. PCR has emerged as the most powerful diagnostic tool for the detection, quantification and genotyping of active HCV RNA in the blood.

In the present study, over 4000 blood samples were randomly collected from individuals visiting different clinical laboratories in Lahore, Pakistan. These samples were screened through confirmatory nested PCR qualitative assay to determine the percent prevalence of active HCV in various subjects. To the best of our knowledge, this is the first comprehensive report concerning the prevalence of active HCV in Pakistan’s 2^nd^ largest city and its surroundings, with a statistically significant number of samples. The data was also correlated and categorized in terms of gender and age groups. This report will thus provide the HCV prevalence data for further meta-analysis that can help to devise strategies by the health policy makers for the control of hepatitis C disease.

## Results

Out of 4246 blood samples, 1914 were collected from male and 2332 from female subjects. Moreover, all the individual samples were categorized into six age groups. The PCR results revealed that 210 (4.9%) individuals had active HCV infection (Table 1). Gender-wise prevalence of active HCV infection was estimated to be 5.27% in male (101 positive out of 1914 samples) and 4.67% in female subjects (109 positive out of 2332 samples), respectively. Although the probability trends were slightly higher among males of all age groups than females, statistically there was no significant difference in gender with OR = 1.10 CI = (0.83-1.46), *p* >0.05, as elaborated in Table 1.

**Table 1:** Prevalence of active hepatitis C virus in general public of Lahore

**Age groups**	**Total samples**	**Male + / -**	**Female + / -**	**Probabilities**^**a**^		***p *** **value**^**c**^	**Odd ratio (95% CI)**	**Overall prevalence (%)**
				**Male**	**Female**			
9-19	458	9/211	6/232	0.0343	0.0313	--	--	3.3
20-29	891	31/399	38/423	0.0810	0.0741	0.002	2.48	7.7
							(1.40-4.38)	
30-39	625	24/258	16/327	0.0672	0.0614	0.023	2.03	6.7
							(1.10-3.71)	
40-49	654	14/296	19/325	0.0529	0.0483	0.155	1.57	5.1
							(0.84-2.93)	
50-59	730	11/298	15/406	0.0375	0.0342	0.779	1.10	3.6
							(0.57-2.09)	
> 59	888	12/351	15/510	0.0321	0.0292	0.831	0.93	3.0
							(0.49-1.77)	
**Total**	**4246**	**101/1813**	**109/2223**	**0.0508**^**b**^	**0.0464**^**b**^	**--**	**--**	**4.9**

^a^Represents the estimated probabilities of HCV prevalence in male and female in different age groups calculated using the inverse transformation of logistic regression model. ^b^Average probability of six age groups of both genders. ^c^The goodness of fit tests Pearson, deviance, and Hosmer-Lemeshow for the model have *p* values ranging from 0.312 to 0.724 indicating the logistic regression model fit adequately HCV prevalence data. ^d^CI (Confidence Interval) for male gender was 1.10 (0.83-1.46) with *p* value 0.497.

Distribution of positive & negative in gender as well as age groups based criteria.

**Figure 1:**
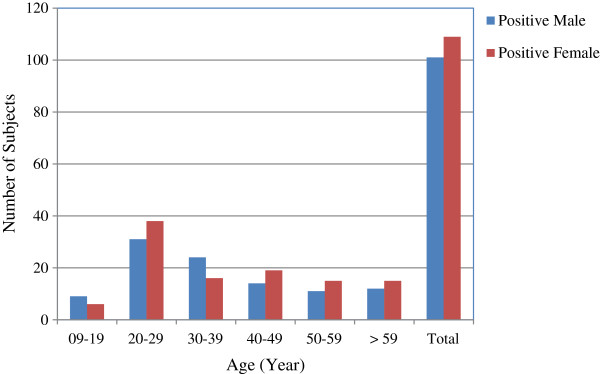
Prevalence of HCV positive samples in male/female population in different age groups.

Substantial differences were observed considering the age group criterion. Out of the 458 individuals tested in ages ranging from 9–19 years (both male and female), only 15 (3.3%) were HCV positive. The highest numbers of positive individuals (69) with 7.7% prevalence were among the tested subjects (891) in the age group 20–29 years (Table 1 and Figure 1). Statistically, the odd of prevalence of HCV in individuals in age group 20–29 years was 14.8% higher than the individuals in age group 9–19 years (OR = 2.48,CI = (1.40-4.38), *p* < 0.05), as illustrated in Table 1.

In the age group 30–39 years, HCV prevalence, though slightly less than that found for the 20–29 year group, was still significantly higher (10.3%) as compared to age group 09–19 years (OR = 2.03, CI = (1.10-3.71). Subsequently, a decreasing trend of HCV prevalence was observed with increasing age of subjects above 40 years. The lowest prevalence (3%) of active HCV was observed in the age group 59 years and above. Overall active HCV prevalence among all the tested samples was estimated to be 4.9% and the risk of HCV prevalence was significantly higher in the middle age groups (20–29 & 30–39 years).

## Discussion

In Pakistan, ~6% population is suspected to be infected with HCV [6,7,25] and HCV prevalence data published so far is highly variable. In most of the studies conducted so far, either the number of samples reported are too small to draw any solid conclusion or the methodological differences have made it impossible to conduct a formal meta-analysis to determine accurate prevalence estimates [7]. Among all the published reports, 99% of the data originated from erroneous non-PCR qualitative screening methodologies, mostly based on the detection of anti-HCV antibodies. Active HCV prevalence estimated during our research (4.9%) is lower than that reported by Aslam et al. [24] (6.7% based on 488 samples from the general population of Lahore). Similarly, the current estimate is much lower when compared with a nationwide data surveillance study conducted through an ELISA blood screen by Qureshi, et al. [12], who reported 6.8% HCV prevalence in Lahore based general public. Our estimates were, however, much higher than those of another study in which 203 blood samples from staff and students of University of the Punjab, Lahore, were assayed through ELISA by Tanvir et al. in 2008 [26]. In yet another series of research conducted in pediatric population by Khan et al. [22], Parker et al. [23] and Hyder et al. [27] HCV prevalence was reported as 4.09%, 1.3% and 0.58%, respectively, which is again lower as in comparison to our reported results (Table 2).

**Table 2:** Previous HCV prevalence data among different communities of Lahore

**Ref. #**	**Population type**	**Author**	**Methods**	**Population size**	**HCV (%)**
[24]	General population	Aslam et al. 2001	ICT	488	6.70
[26]	General population	Tanvir et al. 2008	ICT	203	1.48
[12]	General population	Qureshi et al. 2010	ELISA	--^**a**^	6.80
[11]	General population	Bosan et al. 2010	ICT, Lattix	1501^**b**^	2.1-13.5
[22]	Pediatric population	Khan et al. 1996	EIA, RIBA	538	4.09
[23]	Pediatric population	Parker et al. 1999	ELISA	538	1.30
[27]	Pediatric population	Hyder et al. 2001	ELISA	171	0.58
[28]	Pregnant women	Zafar et al. 2001	PCR	300	4.00
[11]		Bosan et al. 2010	ELISA	4108^**b**^	6.8-7.3
[29]	Blood donors	Chaudhary et al. 2005	ELISA	890	6.06
[11]	Blood donors	Bosan et al. 2010	EIA, ELISA, ICT	32326^**b**^	4.1-6
[21]	Blood donors	Akhtar et al. 2013	ELISA	245	17.78
[31]	Hemophilia	Malik et al. 2006	ELISA	100	56
[11]	Other high risk groups	Bosan et al. 2010	ELISA	412^**b**^	19-56

^**a**^In this national survey, samples collected from Lahore region were not mentioned.

^**b**^Data from a number of locally published reports, reviewed by Bosan et al. 2010 [11], have been combined.

The only PCR-based HCV active prevalence study conducted in Lahore was reported by Zafar et al. [28] for a cohort of pregnant women. Out of 300 screened samples, 4% were found to be positive. Although this figure is relatively close to our figure (4.9%) the number of samples tested and the study subjects were less broad. Moreover, our active HCV prevalence estimate in general public of Lahore city is much lower than the values reported by Chaudhary et al. [29] and Akhtar et al. [21] among blood donors in Lahore, which revealed HCV prevalence as 6.06% and 17.78%, respectively; indicating highly variable results (Table 2). Both studies were conducted using ELISA as a screening tool. HCV prevalence in IDUs [30] and haemophilia patients [31], reported as 88% and 56% respectively, are much higher in comparison with our results.

Gender-wise HCV prevalence revealed no significant difference [OR = 1.10 CI = (0.83-1.46), *p* > 0.05] in male and female populations, as detailed in Table 1. Our study is in agreement with the previous country-wide as well as Lahore based surveys [12,20,32]. It is, however, in contrast with a recent study showing female to male ratio of 1:16.5 (Table 2) presumably due to the subjects were restricted only to blood donors and ELISA was used as a diagnostic tool [21].

Considering the age group criterion, significant differences were observed in the prevalence of HCV in both genders. The highest prevalence 7.7% and 6.4% were observed in age groups 20–29 and 30–39 years, respectively; with odds of prevalence of 14.8% (OR = 2.48, CI = (1.40-4.38), *p* < 0.05) and 10.3% (OR = 2.03, CI = (1.10-3.71), respectively. Both of these values are higher compared to the age group 9–19 years. In age groups above 40 years (40–49, 50–59 and >59 years), a decreasing trend of active HCV prevalence was observed (Table 1). These results are in agreement with the previous studies particularly those conducted in Lahore city revealing the higher risk of HCV prevalence in middle aged groups (20–40 years) [20,24,33,34]. The high prevalence of HCV in middle aged groups can be correlated to more exposure of HCV infection and other risk factors such as non-blood transfusions, widespread reuse of syringes, and a range of other high-risk traditional practices.

## Conclusions

Using the PCR based diagnostic assay for 4246 blood samples, the overall prevalence of active HCV was estimated as 4.9% in general public of Lahore. No significant differences in male and female genders were observed. However, HCV prevalence varied in different age groups. It was least prevalent in age groups 9–19 and above 59 years. However, middle aged populations, especially 20–29 and 30–39 year individuals were observed at higher risk of hepatitis C ailments with 7.7% and 6.4% active HCV prevalence, respectively. This report will provide the active HCV prevalence data for further meta-analysis, which can be helpful to health policy makers to devise strategies for the control of hepatitis C disease in Lahore in particular and in Pakistan in general. From the results of the present study, future PCR-based studies will result in lowering the previously reported estimates (i.e. 6%) of prevalence of HCV in Pakistan.

## Methodology

### Collection of blood samples

In this study, 4246 blood samples were collected randomly from individuals visiting different clinical laboratories of Lahore between 2010 and 2012. As the study was designed to represent the general public HCV prevalence, the samples were collected randomly from individuals who visited laboratories for any purpose such as some clinical test, sample submission, report collection or blood screen etc. Samples were collected from both genders having ages ranging from 9 to > 59 years. Informed consent was taken from every individual being tested and approval was obtained from institutional ethical review committee. History of individuals was recorded in the form of questionnaires. Serum from each of these blood samples were separated by centrifugation at 4000 rpm for 5 min. Each sample was properly labeled and stored at -20°C until (on every coming Monday) it was shifted to National Institute for Biotechnology & Genetic Engineering (NIBGE) Faisalabad for PCR diagnostic assay. Besides the gender groups, samples were also categorized into six age groups to determine the prevalence in each age group.

### Viral RNA extraction and cDNA formation

RNA was extracted from these samples using the Qiamp Viral RNA extraction kit (Qiagen, USA) according to manufacturer’s instruction. Serum sample (140 μl) was used to extract RNA that was eluted in 60 μl elution buffer supplied with kit. RNA was used in making cDNA immediately after extracting RNA or stored at -80°C for further use. cDNA was prepared in a total reaction volume of 20 μl containing 10 μl of RNA extracted from each sample. For cDNA formation, First Strand cDNA synthesis kit (Thermo scientific, USA) was used. Briefly, the extracted RNA was mixed with 20 picomol of gene specific primer (5′ GTGCACGGTCTACGAGACCT 3′) and 200 units Moloney Murine Leukemia Virus (M-MuLV) reverse transcriptase and incubated at 42°C for 60 min with light shaking. After incubation, cDNA was properly labeled and stored at -20°C for PCR amplification.

### Polymerase chain reaction amplification of cDNA

Amplification of cDNA was performed according to Petrelli [35]. A reaction mixture of 50 μl containing 5 μl of cDNA, 0.2 mM dNTPs, 2 mM MgCl_2_, 0.5 μM of forward primer, 0.5 μM of reverse primer, 2.5 U of Taq DNA Polymerase and 1X Taq polymerase Buffer (75 mM Tris–HCl pH 8.8, 20 mM (NH_4_)_2_SO_4_, 0.01% Tween) [Fermentas] was prepared and amplification was carried out in a thermal cycler (Biorad). For amplification of first round of PCR, conditions used were 94°C for 2 min followed by 30 cycles with the temperature profile 94°C for 30 sec, 55°C for 30 sec and 72°C for 30 sec and final extension was performed for 2 min at 72°C. The PCR products were used as template for nested PCR amplification. The temperature conditions were the same as in the first round of PCR except the primers used, which were complementary to the PCR product of first round PCR [36].

### Analysis of PCR product

The amplified PCR products of nested PCR were run on 1% agarose gel prepared in 1X TBE (Trizma base, boric acid) buffer. Ethidium bromide dye (3 μL, 1% w/v) was also added in order to make the bands visible under UV light. The samples were loaded into the well by mixing with bromophenol blue and gel was run under constant voltage of 80 until the dye reached the other end of the gel. The bands were visualized under UV light. The sensitivity of the PCR based qualitative assay was noticed around 200 IU per ml of the sample.

### Statistical analysis

All of the PCR based HCV qualitative data were statistically analyzed using Minitab version 16.2 for Windows. Binary Logistic regression was performed in order to examine the prevalence of HCV associated with sex and age. Statistical tests of the regression estimates or odds ratios (OR) were based on Wald statistics. The Hosmer-Lemeshow statistics has suggested that the logistic regression model fit the data adequately and 95% confidence intervals for odd ratios were calculated. A *p*-value < 0.05 was defined as statistically significant.

## Abbreviations

HCV: Hepatitis C virus; IDUs: Injection drug users; ICT: Immunochromatographic; EIA: Enzyme immunoassay; RIBA: Recombinant strip immunosorbent assay; ELISA: Enzyme linked immunosorbent assay; RT-PCR: Reverse transcriptase polymerase chain reaction; CDNA: Complimentary DNA; WHO: World Health Organization; OR: Odd ratio; CI: Confidence interval.

## Competing interests

The authors declare that they have no competing interests.

## Authors’ contributions

MIA carried out mainly this prevalence studies. He collected the samples, got the consent of subjects and performed PCR based qualitative assays. MR shared the cost of research, helped in design of study and contributed in manuscript preparation. MUH conducted the statistical analysis and helped in getting the samples. MI mainly supervised this study and prepared the manuscript. All authors read and approved the final manuscript.

## Authors’ information

^1^MIA PhD student, MR Senior Scientist & MI Principal Scientist/Group Leader at Drug Discovery and Structural Biology, Health Biotechnology Division, National Institute for Biotechnology and Genetic Engineering (NIBGE), Faisalabad-38000, Pakistan. ^2^MUH is currently MS student at Department of Statistics, Stockholm University, SE - 106 91, Stockholm, Sweden.
